# Comparison of 1064‐nm Picosecond and Q‐Switched Neodymium‐Doped Yttrium Aluminum Garnet Lasers for Melasma in Asian Women: A Randomized Double‐Blind Split‐Face Trial

**DOI:** 10.1111/jocd.71008

**Published:** 2026-06-23

**Authors:** Ying Wu, Qixuan Wang, Jiayi Feng, Zehua Chen, Lvping Huang, Yongqiang Feng, Xiaojing Li

**Affiliations:** ^1^ Department of Plastic Surgery The First Affiliated Hospital of Anhui Medical University Hefei China; ^2^ Laser Aesthetic Center, Plastic Surgery Hospital Peking Union Medical College & Chinese Academy of Medical Sciences Beijing China; ^3^ Department of Plastic Surgery Shenzhen Hospital of Southern Medical University Shenzhen Guangdong China

**Keywords:** melasma, Nd:YAG laser, picosecond, Q‐switched, randomized trial, split‐face trial

## Abstract

**Background:**

Melasma is a chronic, relapsing facial hyperpigmentation that remains challenging to treat in medium‐to‐dark phototypes.

**Aims:**

To compare the efficacy, safety, tolerability, and recurrence of 1064‐nm picosecond neodymium‐doped yttrium aluminum garnet laser treatment versus 1064‐nm Q‐switched neodymium‐doped yttrium aluminum garnet laser treatment in Asian women with melasma.

**Methods:**

In this prospective, randomized, double‐blind, split‐face trial, 16 Asian women with Fitzpatrick skin types III–IV received three treatment sessions at 4‐week intervals. Laser allocation was randomized by hemi‐face, and outcome assessors were blinded. The primary endpoint was the hemi‐modified Melasma Area and Severity Index (hemi‐mMASI), assessed at baseline and at 1, 2, 3, and 6 months after the final treatment session. Secondary outcomes included the Global Aesthetic Improvement Scale (GAIS), recurrence, and pain intensity measured using a visual analog scale.

**Results:**

Both 1064‐nm picosecond Nd:YAG and 1064‐nm Q‐switched Nd:YAG laser treatments were associated with significant reductions in hemi‐mMASI over time. No statistically significant between‐treatment differences in hemi‐mMASI or GAIS were detected during follow‐up. Pain scores after the first treatment session were significantly lower on the picosecond‐treated side. Recurrence rates at 6 months did not differ significantly between treatments.

**Conclusions:**

In Asian women with Fitzpatrick skin types III–IV, both 1064‐nm picosecond Nd:YAG and 1064‐nm Q‐switched Nd:YAG laser treatments were associated with clinical improvement in melasma. No statistically significant between‐treatment differences in hemi‐mMASI or GAIS were detected in this small split‐face trial, whereas picosecond treatment was associated with lower pain scores after the first treatment session.

**Trial Registration:**

Chinese Clinical Trial Registry registration number: ChiCTR2000030886

## 1 Introduction

Melasma is a common, acquired, benign disorder of pigmentation characterized by symmetric brown‐to‐gray macules and patches, most often on the face. It affects individuals with medium‐to‐dark phototypes (especially Fitzpatrick III–IV) and is particularly prevalent in Asian populations [1, 2, 3].

Topical hydroquinone (HQ; 1,4‐dihydroxybenzene) remains a first‐line therapy because it suppresses melanogenesis mainly through tyrosinase inhibition, and it is most effective for epidermal‐predominant disease. Oral tranexamic acid, via anti‐inflammatory and anti‐oxidative pathways, has also shown meaningful benefit in many patients, especially when used as an adjunct therapy [2, 4]. Among energy‐based approaches, intense pulsed light (IPL), fractional lasers, and ablative CO_2_ platforms can produce modest improvements, but the 1064‐nm Q‐switched Neodymium‐Doped Yttrium Aluminum Garnet (Nd:YAG) laser is widely regarded as one of the most effective light‐based options, particularly for mixed or dermal melasma and for moderate‐to‐severe disease [5, 6].

However, in Asian patients this modality is still limited by post‐inflammatory hyperpigmentation and frequent relapse. For example, Hofbauer et al. treated 20 Brazilian women with weekly 1064‐nm Q‐switched Nd:YAG sessions (10 treatments total), observing clear short‐term modified Melasma Area and Severity Index (mMASI) improvement but an 81% recurrence rate within 3 months [7]. Because melasma pathogenesis is multifactorial and not fully clarified, durable disease control remains challenging [2, 3].

Picosecond lasers have recently been introduced for pigmentary disorders. Compared with nanosecond Q‐switched devices, they can create smaller pigment particles that are more readily cleared by macrophages, potentially lowering the risk of PIH [6, 8]. In a split‐face trial, Chalermchai et al. combined a fractional 1064‐nm picosecond laser with 4% HQ and achieved a significantly greater reduction in mMASI than HQ alone, supporting the therapeutic value of 1064‐nm picosecond treatment in melasma [9].

Accordingly, we designed a randomized split‐face trial to compare the efficacy, safety, tolerability, and recurrence profiles of 1064‐nm picosecond Nd:YAG laser treatment and 1064‐nm Q‐switched Nd:YAG laser treatment in patients with melasma.

## 2 Materials and Methods

### 2.1 Study Design and Participants

This study was a prospective, randomized, double‐blind, split‐face clinical trial designed to compare 1064‐nm picosecond Nd:YAG laser and 1064‐nm Q‐switched Nd:YAG laser for the treatment of melasma. We evaluated efficacy, safety, and recurrence. The protocol was approved by the Ethics Committee of Plastic Surgery Hospital, Chinese Academy of Medical Sciences (approval No. [2022] Registration No. 40; approval date: March 7, 2022). The trial was registered prior to enrollment. All procedures complied with the Declaration of Helsinki. Written informed consent was obtained from all participants.

Inclusion criteria: Patients diagnosed with melasma and agreed to comply with the study procedures and follow‐up visits were included. Exclusion criteria: patients were excluded if they had a history of photosensitivity disorders; were pregnant or lactating; had significant hepatic or renal impairment or other conditions that would contraindicate laser therapy (e.g., active thrombotic disease or recent myocardial infarction); had coexisting focal facial pigmented lesions that could confound outcome assessment (such as lentigines, nevus of Ota, or nevus spilus); had received systemic, topical, or light/energy‐based therapies for melasma within the prior 6 months; or were judged by the investigator to be otherwise unsuitable for participation.

**FIGURE 1:**
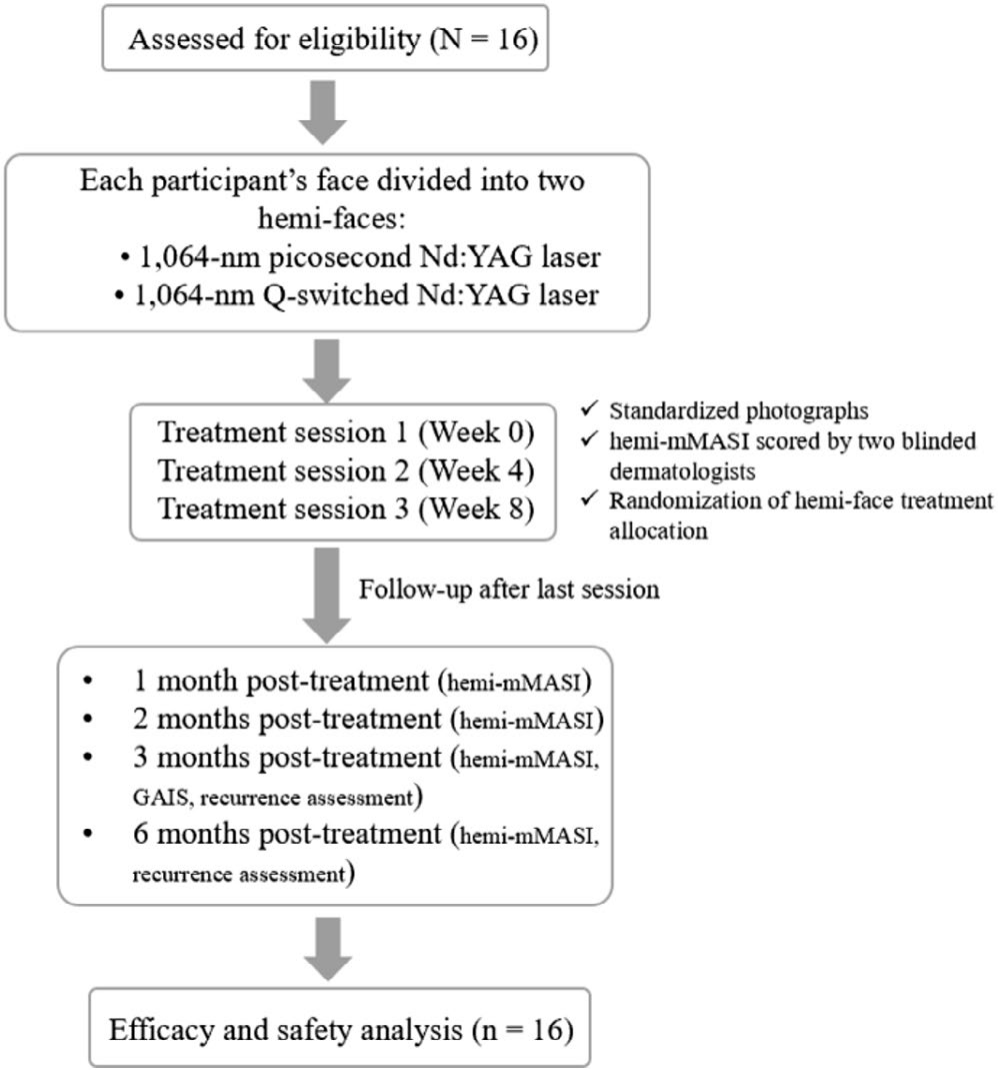
Study flow diagram and follow‐up schedule. Sixteen patients with facial melasma were assessed for eligibility, completed three split‐face treatment sessions with 1064‐nm picosecond Nd:YAG and 1064‐nm Q‐switched Nd:YAG laser treatments, completed the 6‐month follow‐up, and were included in the efficacy and safety analyses.

Each patient's face was divided into left and right hemi‐faces; allocation of picosecond versus Q‐switched laser treatment to each hemi‐face was determined by random number table. Outcome assessors (two dermatologists) were blinded to treatment allocation. The study flow diagram and follow‐up schedule are shown in Figure 1.

### 2.2 Treatment Protocol

The hemi‐face assigned to picosecond treatment was treated using a commercially available 1064‐nm picosecond Nd:YAG laser system: PicoWay laser system (Candela Corporation, Wayland, MA, USA). Treatment was delivered using a zoom handpiece with a 6‐mm spot size, fluence of 0.5–0.7 J/cm^2^, 10 Hz, and approximately 1000 pulses, followed by the Resolve 1064‐nm handpiece at approximately 0.9 mJ, 10 Hz, and approximately 1000 pulses, resulting in approximately 2000 pulses per session.

The contralateral hemi‐face was treated using a commercially available 1064‐nm Q‐switched Nd:YAG laser system: Revlite SI Q‐switched Nd:YAG laser system (Cynosure Inc., Westford, MA, USA). Two parameter sets were used: an 8‐mm spot size at 2.0–2.3 J/cm^2^ and 10 Hz for approximately 1000 pulses, followed by a 6‐mm spot size at 2.5–3.0 J/cm^2^ and 10 Hz for approximately 1000 pulses, resulting in approximately 2000 pulses per session. Treatment parameters were adjusted by clinicians to achieve the treatment endpoint of mild erythema while maintaining patient tolerance. The treatment course comprised three sessions at 4‐week intervals. Patients were instructed to avoid other melasma treatments during the study and to practice strict photoprotection with sunscreen SPF ≥ 30 and physical measures.

Device names and manufacturers are provided solely to ensure reproducibility of the treatment parameters and do not imply endorsement of, or preference for, any specific commercial laser system.

### 2.3 Outcome Assessments

Assessments of hemi‐modified Melasma Area and Severity Index (hemi‐mMASI) were performed at the following time points: baseline, 1, 2, 3 and 6 months after completion of the last treatment. Standardized facial photographs were obtained at each visit under consistent lighting, camera settings, patient positioning, and background conditions. Written informed consent for publication of clinical images was obtained from the participant.

Primary outcome is hemi‐mMASI, calculated as follows: hemi‐mMASI = 0.15 × A_forehead × D_forehead + 0.3 × A_cheek × D_cheek + 0.05 × A_nasolabial × D_nasolabial (where A = area score, D = darkness score). Two blinded dermatologists independently scored the hemi‐mMASI on standardized images; the mean of two scores was used.

Secondary outcomes included the Global Aesthetic Improvement Scale (GAIS), a 5‐point ordinal scale recorded at 3 months post‐treatment (1 = much improved; 2 = improved; 3 = somewhat improved; 4 = no change; 5 = worse).

Percentage reduction in hemi‐mMASI was calculated as $\frac{\text{baseline mMASI}-\text{follow}-\mathrm{up}\;\text{mMASI}}{\text{baseline mMASI}}\times 100\%$. Treatment response was categorized as follows: near‐complete clearance (≥ 80% reduction), marked (≥ 50% to < 80%), improved (≥ 20% to < 50%), and ineffective (< 20%). The efficacy rate was defined as the proportion of hemi‐faces achieving a near‐complete clearance or marked response, that is, $\frac{\text{near}-\text{complete clearance}+\text{marked}}{n}\times 100\%$.

Recurrence was predefined as an increase in hemi‐mMASI of ≥ 20% relative to the score recorded immediately before the third treatment session. This reference point was selected because it represented the last on‐treatment assessment before completion of the treatment course and provided a consistent reference point for both hemi‐faces before the final treatment session. Recurrence rate was calculated per hemi‐face as: $\frac{\text{number of recurrent hemi}-\text{faces}}{\text{total number of hemi}-\text{faces}}\times 100\%$. To assess the robustness of this definition, a nadir‐based sensitivity analysis was additionally performed. In this sensitivity analysis, 6‐month recurrence was defined as a ≥ 20% increase from the lowest post‐treatment hemi‐mMASI score observed during the 1‐, 2‐, or 3‐month follow‐up visits.

Safety endpoints included immediate pain intensity assessed using a 0–10 visual analog scale (VAS), recorded immediately after the first treatment session for each hemi‐face.

### 2.4 Statistical Analysis

Statistical analyses and visualization were performed using SPSS (v.27) and R (v.4.5.1). Continuous variables were tested for normality (Shapiro–Wilk). Paired *t*‐test was used for normally distributed paired comparisons; Wilcoxon signed‐rank test otherwise. For time × treatment interaction, two‐way repeated‐measures ANOVA was used, with Greenhouse–Geisser correction if sphericity was violated. Categorical outcomes (efficacy categories, recurrence) were analyzed with McNemar's or paired binomial tests. *p* < 0.05 was considered statistically significant.

Because this was a paired split‐face study, the sample size was estimated using a paired‐design formula. Based on preliminary estimates, an expected paired difference of 0.57 in hemi‐mMASI and a standard deviation of paired differences of 0.70 were used. Using a paired‐design superiority framework with *α* = 0.05 and 90% power, 13 participants were required. After allowing for an anticipated dropout rate of approximately 20%, the target sample size was set at 16 participants.

Inter‐rater reliability for hemi‐mMASI scoring was assessed across all hemi‐face assessments from scheduled visits using the intraclass correlation coefficient (ICC). A two‐way mixed‐effects, absolute‐agreement, average‐measures model was used because the mean of the two blinded dermatologists' scores was used for the primary efficacy analysis.

## 3 Results and Discussion

### 3.1 Participant Characteristics

Sixteen participants were enrolled, and all completed the treatment course and follow‐up visits. Therefore, 16 participants (32 hemi‐faces) were included in the efficacy analysis, and the same cohort was evaluated for safety outcomes. All participants were female. Baseline demographic and clinical characteristics are summarized in Table 1. The mean age at enrollment was 43.6 ± 7.7 years, and the mean duration of melasma was 7.6 ± 4.1 years. Fitzpatrick skin types were type III in 5 patients (31%) and type IV in 11 patients (69%). Most patients had a history of pregnancy and reported at least one potential aggravating factor for melasma, whereas alcohol intake and active smoking were rare (Table 1). Inter‐rater reliability for hemi‐mMASI scoring was good. The ICC was 0.870 (95% CI, 0.795–0.913; *p* < 0.001), supporting the consistency of the blinded assessments.

**TABLE 1:** Baseline demographic and clinical characteristics (*n* = 16).

Characteristics	Values
*n*	16
Sex	Female 16 (100%)
Mean age (mean ± SD), year	43.6 ± 7.7
Mean melasma duration (±SD), year	7.6 ± 4.1
Fitzpatrick skin type, *n* (%)	
III	5 (31%)
IV	11 (69%)
Pregnancy history, *n* (%)	
Yes	14 (88%)
No	2 (12%)
Any factors affecting melasma, *n* (%)	
Yes	12 (75%)
No	4 (25%)
Lifestyle, *n* (%)	
Alcohol intake	1 (6%)
Smoking	0 (0%)
Outdoor worker	0 (0%)
Daily sunscreen use, *n* (%)	6 (38%)
Oral contraception, *n* (%)	2 (13%)
Liver disease, *n* (%)	0 (0%)
Sleep problems, *n* (%)	13 (81%)

Abbreviation: SD, standard deviation.

### 3.2 Changes in Hemi‐mMASI Over Time

**FIGURE 2:**
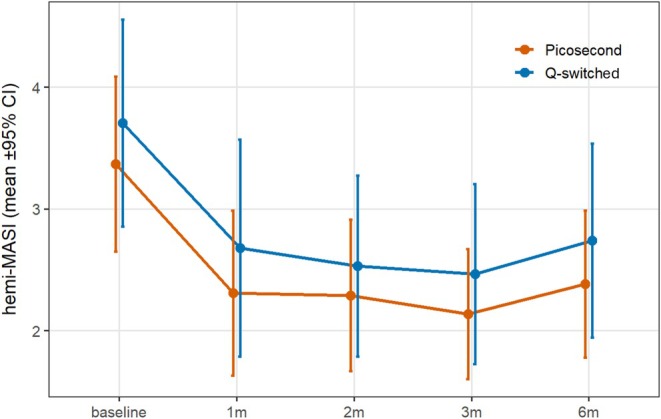
Changes in hemi‐modified Melasma Area and Severity Index (hemi‐mMASI) over time. Mean hemi‐mMASI scores (±95% CI) for hemi‐faces treated with 1064‐nm picosecond Nd:YAG and 1064‐nm Q‐switched Nd:YAG laser treatments from baseline to 6 months after the final treatment. Both treatment modalities showed significant reductions over time (*p* < 0.001 for the main effect of Time), with no significant main effect of Treatment modality or Time × Treatment interaction.

**FIGURE 3:**
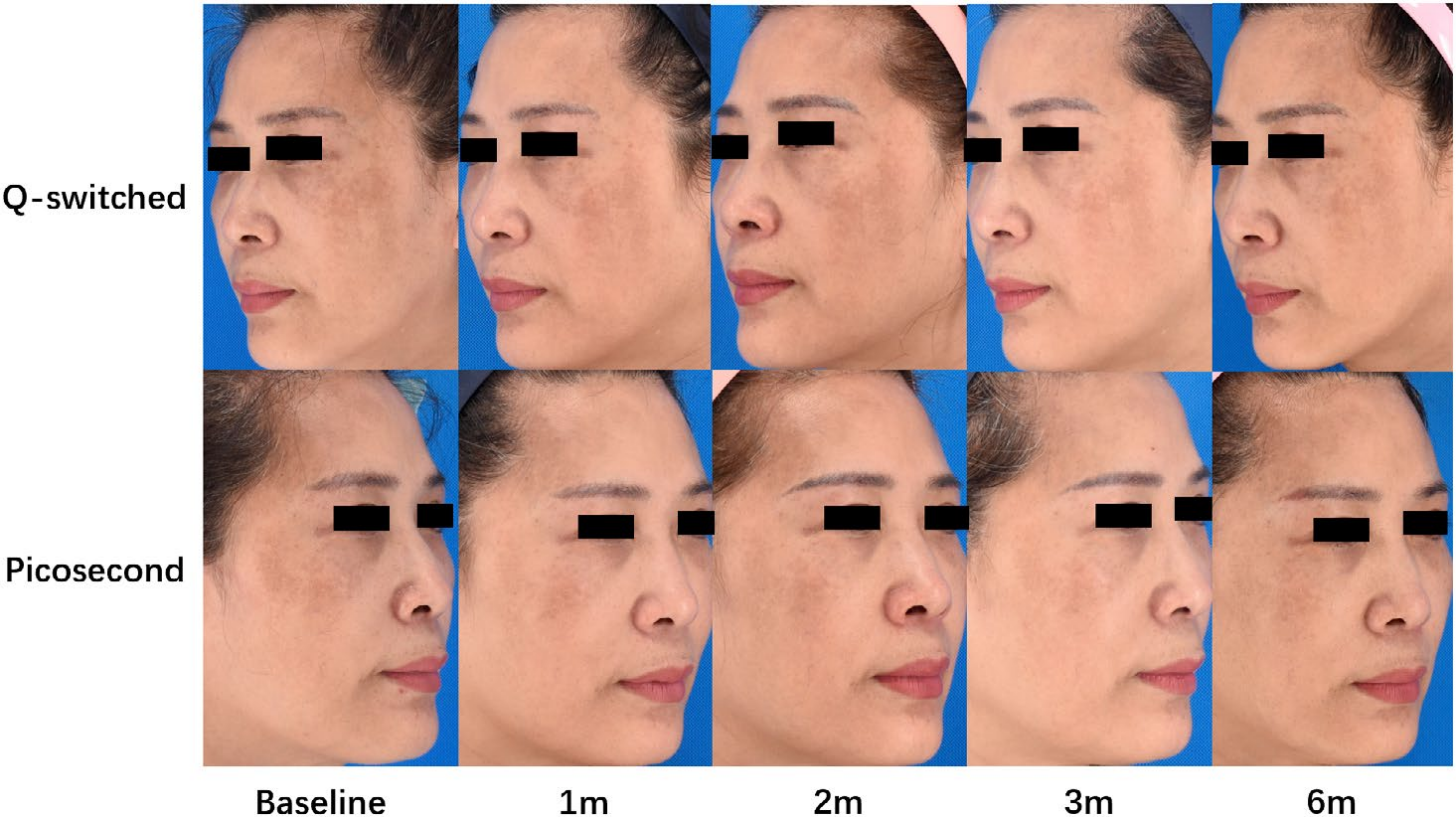
Representative clinical photographs showing longitudinal changes after split‐face laser treatment. The upper row shows the hemi‐face treated with the 1064‐nm Q‐switched Nd:YAG laser, and the lower row shows the hemi‐face treated with the 1064‐nm picosecond Nd:YAG laser. Standardized photographs were obtained at baseline and at 1, 2, 3, and 6 months after the final treatment session. In this representative participant, hemi‐mMASI scores decreased on both sides during follow‐up, with the lowest scores observed around 3 months and a partial rebound at 6 months. Identifying features were masked, and written informed consent for publication was obtained.

At baseline, mean hemi‐mMASI scores were 3.37 ± 1.35 for picosecond‐treated hemi‐faces and 3.70 ± 1.60 for Q‐switched‐treated hemi‐faces, with no statistically significant between‐treatment difference. Both treatment modalities showed reductions in hemi‐mMASI during follow‐up. At 3 months after the final treatment session, mean hemi‐mMASI scores decreased to 2.14 ± 1.00 for picosecond‐treated hemi‐faces and 2.46 ± 1.39 for Q‐switched‐treated hemi‐faces. At 6 months, the corresponding scores were 2.38 ± 1.13 and 2.74 ± 1.49, respectively, indicating partial rebound from the 3‐month visit while remaining below baseline levels. Paired between‐treatment differences were estimated to provide a measure of uncertainty. The picosecond‐treated hemi‐faces showed numerically lower hemi‐mMASI scores than the Q‐switched‐treated hemi‐faces at all evaluated time points; however, no statistically significant between‐treatment difference was detected, and the 95% confidence intervals for paired differences crossed zero (Figure 2, Table 2). Representative anonymized clinical photographs are provided in Figure 3.

**TABLE 2:** Efficacy comparison by hemi‐mMASI (mean ± SD).

Time	Picosecond laser (mean ± SD)	Q‐switched laser (mean ± SD)	Paired difference, picosecond−Q‐switched, mean (95% CI)	*p*
Baseline	3.37 ± 1.35	3.70 ± 1.60	−0.34 (−0.93 to 0.26)	0.249
1 m	2.31 ± 1.27	2.68 ± 1.67	−0.37 (−0.96 to 0.22)^a^	0.361
2 m	2.29 ± 1.17	2.53 ± 1.39	−0.24 (−0.77 to 0.29)	0.351
3 m	2.14 ± 1.00	2.46 ± 1.39	−0.33 (−0.88 to 0.22)	0.224
6 m	2.38 ± 1.13	2.74 ± 1.49	−0.36 (−0.82 to 0.11)	0.123

*Note:* Paired differences were calculated as picosecond‐treated hemi‐face minus Q‐switched‐treated hemi‐face. Negative values indicate numerically lower hemi‐mMASI scores on the picosecond‐treated side. *p* values are for paired between‐treatment comparisons at each time point. Paired *t*‐tests were used for normally distributed paired differences; the Wilcoxon signed‐rank test was used for the 1‐month comparison because the paired differences were not normally distributed.

Abbreviations: CI, confidence interval; m, months; SD, standard deviation.

^a^ The 95% CI for the 1‐month paired mean difference is presented descriptively.

To further evaluate time effects and between‐treatment differences (*n* = 16), a two‐way repeated‐measures ANOVA was performed with within‐subject factors Time (baseline, 1, 2, 3 and 6 months) and Treatment modality (picosecond vs. Q‐switched). There was a highly significant main effect of Time (*p* < 0.001), confirming that hemi‐mMASI decreased significantly during follow‐up in both treatment modalities. In contrast, the main effect of Treatment modality was not significant (*p* = 0.187), indicating no overall difference in mean hemi‐mMASI between the picosecond and Q‐switched sides. The Time × Treatment interaction was also not significant, indicating that no statistically significant difference in longitudinal change was detected between treatment modalities. Results corrected for violations of sphericity using the Greenhouse–Geisser and Huynh–Feldt methods were consistent with the unadjusted analysis. Collectively, these results suggest that the clinical improvement in pigmentation was driven primarily by treatment over time rather than by a statistically significant difference between laser modalities.

### 3.3 Categorical Efficacy Based on Percentage Hemi‐mMASI Reduction

Clinical responses were classified according to percentage reduction in hemi‐mMASI: near‐complete clearance (≥ 80%), marked response (≥ 50% to < 80%), improvement (≥ 20% to < 50%), and ineffective response (< 20%). At 1 month, the picosecond‐treated hemi‐faces showed 4 marked responses, 6 improved responses, and 6 ineffective responses, corresponding to an efficacy rate of 25.0%. The Q‐switched‐treated hemi‐faces showed 6 marked responses, 5 improved responses, and 5 ineffective responses, corresponding to an efficacy rate of 37.5%. At 2 months, both treatment groups showed identical response distributions, with 5 marked, 7 improved, and 4 ineffective responses in each group. At 3 months, the picosecond‐treated hemi‐faces showed 5 marked, 7 improved, and 4 ineffective responses, whereas the Q‐switched‐treated hemi‐faces showed 7 marked, 4 improved, and 5 ineffective responses. At 6 months, the number of marked responders decreased to 3 in the picosecond‐treated hemi‐faces and 4 in the Q‐switched‐treated hemi‐faces, corresponding to efficacy rates of 18.8% and 25.0%, respectively. No statistically significant between‐treatment difference in categorical response distribution was detected over time (Table 3).

**TABLE 3:** Distribution of treatment response by hemi‐face at 1, 2, 3, and 6 months for picosecond Nd:YAG and Q‐switched Nd:YAG laser treatments (*n* = 16 hemi‐faces per side).

Time	Treatment	Near‐complete clearance	Marked	Improved	Ineffective
1 m	Picosecond	0	4	6	6
1 m	Q‐switched	0	6	5	5
2 m	Picosecond	0	5	7	4
2 m	Q‐switched	0	5	7	4
3 m	Picosecond	0	5	7	4
3 m	Q‐switched	0	7	4	5
6 m	Picosecond	0	3	8	5
6 m	Q‐switched	0	4	5	7

*Note:* Treatment responses were defined according to percentage reduction in hemi‐mMASI from baseline: near‐complete clearance (≥ 80%), marked (≥ 50% to < 80%), improved (≥ 20% to < 50%), and ineffective (< 20%).

### 3.4 Global Aesthetic Improvement Scale

GAIS was assessed in all participants (*n* = 16). Subjective outcomes were evaluated using the 5‐point GAIS at 3 months after the final treatment, with lower scores indicating better improvement (1 = much improved, 5 = worse). The mean GAIS was 2.38 ± 0.81 on the picosecond‐treated hemi‐faces and 2.56 ± 0.81 on the Q‐switched‐treated hemi‐faces, corresponding to “mild to moderate improvement” on both sides. No statistically significant between‐treatment difference in GAIS distribution was detected, with most hemi‐faces rated as “much improved” or “improved,” a small number as “unchanged,” and none as “worse.” Wilcoxon signed‐rank testing showed no significant difference between lasers (*Z* = −0.905, *p* = 0.366), suggesting that patient‐perceived improvement was consistent with objective hemi‐mMASI findings.

### 3.5 Recurrence Assessment

Recurrence was defined a priori as a ≥ 20% increase in hemi‐mMASI compared with the last pre‐treatment value, defined as the score recorded immediately before the third treatment session. At 3 months after the treatment course, recurrence was observed in 1 of 16 picosecond‐treated hemi‐faces (6.3%, 95% CI: 0.2%–30.2%) and 1 of 16 Q‐switched‐treated hemi‐faces (6.3%, 95% CI: 0.2%–30.2%). At 6 months, recurrence was observed in 2 of 16 picosecond‐treated hemi‐faces (12.5%, 95% CI: 1.6%–38.3%) and 3 of 16 Q‐switched‐treated hemi‐faces (18.8%, 95% CI: 4.0%–45.6%). Although recurrence was numerically higher on the Q‐switched‐treated side under this predefined definition, the difference was small and not statistically significant. To further assess the robustness of the recurrence findings, we performed a nadir‐based sensitivity analysis, in which 6‐month recurrence was defined as a ≥ 20% increase from the lowest post‐treatment hemi‐mMASI score observed during the 1‐, 2‐, or 3‐month follow‐up visits. Using this definition, 6‐month recurrence was observed in 8 of 16 ps‐treated hemi‐faces (50.0%) and 8 of 16 Q‐switched‐treated hemi‐faces (50.0%). Thus, the nadir‐based definition yielded higher absolute recurrence estimates, as expected, but the overall between‐treatment pattern remained unchanged. Notably, a slight rebound in mean hemi‐mMASI at 6 months compared with 3 months was observed on both sides, consistent with the chronic relapsing course of melasma and underscoring the potential need for maintenance strategies. Recurrence assessments according to predefined and nadir‐based definitions are shown in Table 4.

**TABLE 4:** Recurrence assessments according to predefined and nadir‐based definitions.

Recurrence definition	Timepoint	Picosecond, *n*/*N* (%)	Q‐switched, *n*/*N* (%)
Predefined	3 m	1/16 (6.3%)	1/16 (6.3%)
Predefined	6 m	2/16 (12.5%)	3/16 (18.8%)
Nadir‐based	6 m	8/16 (50.0%)	8/16 (50.0%)

*Note:* The predefined recurrence definition used the hemi‐mMASI score recorded immediately before the third treatment session as the reference. The nadir‐based sensitivity analysis defined 6‐month recurrence as a ≥ 20% increase from the lowest post‐treatment hemi‐mMASI score observed during the 1‐, 2‐, or 3‐month follow‐up visits. Percentages were calculated per hemi‐face.

Abbreviation: m, months.

### 3.6 Safety and Tolerability

All 16 participants completed treatment without discontinuation. No serious treatment‐related adverse events were observed during the study period, suggesting an acceptable short‐ to mid‐term safety profile under conservative parameters in Fitzpatrick III–IV skin. Immediate pain after the first treatment session differed between treatment modalities. The mean VAS score was 3.13 ± 1.82 (range, 0–7) for picosecond‐treated hemi‐faces and 5.31 ± 1.92 (range, 2–8) for Q‐switched‐treated hemi‐faces. Wilcoxon signed‐rank testing demonstrated significantly higher pain scores on the Q‐switched‐treated hemi‐faces (*Z* = −3.07, *p* = 0.002), with 14 of 16 participants reporting higher VAS scores on the Q‐switched‐treated side and only 1 showing the opposite pattern.

These findings indicate that, although no statistically significant between‐treatment difference in hemi‐mMASI improvement was detected, 1064‐nm picosecond Nd:YAG laser treatment was associated with lower immediate pain after the first treatment session. Taken together, both 1064‐nm picosecond Nd:YAG and 1064‐nm Q‐switched Nd:YAG laser treatments were associated with significant reductions in hemi‐mMASI over follow‐up. No statistically significant between‐treatment differences in hemi‐mMASI, GAIS, or recurrence were detected. However, because this study was designed as a paired split‐face comparative study rather than an equivalence or non‐inferiority trial, the absence of statistical significance should not be interpreted as evidence of equivalent efficacy. Our results align with emerging evidence comparing 1064‐nm picosecond and conventional 1064‐nm Q‐switched laser treatments in Asian populations. Hong et al. conducted a prospective split‐face study in Korean patients and found that 1064‐nm picosecond Nd:YAG laser achieved significant mMASI improvement but was not superior to 1064‐nm Q‐switched laser treatment at any follow‐up point [10]. Feng et al. [11] similarly reported no statistically significant efficacy difference between picosecond and Q‐switched 1064‐nm Nd:YAG lasers in a split‐face RCT, although tolerability tended to favor picosecond treatment. In contrast, several studies using picosecond systems at other wavelengths (particularly 755‐nm picosecond alexandrite) have shown faster or greater improvement than 1064‐nm Q‐switched laser [12]. This implies that treatment outcomes may be influenced not only by pulse width but also by wavelength‐dependent melanin absorption.

The favorable safety profile observed in our cohort is also clinically relevant. No serious adverse events occurred, and the conservative treatment settings appeared appropriate for Fitzpatrick III–IV skin. Meanwhile, the pattern of mild rebound and increasing recurrence by 6 months on both sides mirrors the relapsing nature of melasma, and previous studies likewise suggest that laser‐induced improvement may be temporary without appropriate maintenance therapy [13]. Together, these findings suggest that both modalities may provide short‐ to mid‐term clinical improvement under conservative parameters, while picosecond treatment may offer an advantage in immediate procedural comfort.

This study used a randomized split‐face design, allowing each patient to serve as their own control and strengthening the within‐subject comparison between the two lasers. Multiple outcome measures and a six‐month follow‐up enabled evaluation of both early response and mid‐term stability. Nevertheless, several limitations should be acknowledged. The sample size was small; all participants were female and recruited from a single center, and only three treatment sessions were administered. Although the sample size was estimated for the paired split‐face design, the study was not designed as an equivalence or non‐inferiority trial. Therefore, the absence of a statistically significant between‐treatment difference should not be interpreted as proof of equivalent efficacy. Given the small sample size, the possibility of a Type II error cannot be excluded, and larger adequately powered studies are needed to determine whether smaller but clinically relevant between‐treatment differences exist. Pain VAS scores were recorded only after the first treatment session; therefore, they may not fully represent tolerability across the entire three‐session treatment course. Although representative standardized clinical photographs were added to strengthen visual documentation, dermoscopic imaging was not systematically collected at all follow‐up visits and therefore was not included as a formal outcome measure. Future studies should incorporate standardized dermoscopic, colorimetric, or other objective imaging assessments to provide more detailed evaluation of pigmentary changes.

## Author Contributions

Conceptualization: Xiaojing Li, Yongqiang Feng, Lvping Huang and Ying Wu; Methodology: Xiaojing Li, Yongqiang Feng, Jiayi Feng and Ying Wu; investigation: Lvping Huang, Jiayi Feng; data curation: Ying Wu, Yongqiang Feng and Zehua Chen; formal analysis: Qixuan Wang; visualization: Qixuan Wang; writing – original draft: Qixuan Wang and Ying Wu; writing – review and editing: Qixuan Wang, Ying Wu and Yongqiang Feng; supervision: Xiaojing Li and Yongqiang Feng. All authors read and approved the final manuscript.

## Funding

The authors have nothing to report.

## Ethics Statement

This study was approved by the Ethics Committee of Plastic Surgery Hospital, Chinese Academy of Medical Sciences (approval No. [2022] Registration No. 40; approval date: March 7, 2022), and was conducted in accordance with the Declaration of Helsinki.

## Consent

Written informed consent was obtained from all individual participants included in the study.

## Conflicts of Interest

The authors declare no conflicts of interest.

## Data Availability

The data that support the findings of this study are available on request from the corresponding authors. The data are not publicly available due to privacy or ethical restrictions.
